# Novel glucagon-like peptide-1 analogue exhibits potency-driven G-protein biased agonism with promising effects on diabetes and diabetic dry eye syndrome

**DOI:** 10.1080/21655979.2022.2031418

**Published:** 2022-02-19

**Authors:** Yongna Hao, Min Wei, Ning Zhang, Xinying Zhang

**Affiliations:** a Corneal Department, Handan City Eye Hospital, Handan, PR China; b Infection Control Office, Affiliated Hospital of Hebei University, Baoding, PR China

**Keywords:** GLP-1, G-protein, long-lasting diabetes, dry eye syndrome, obesity

## Abstract

Glucagon-like peptide-1 receptor agonists (GLP-1RAs) are considered as effective treatments for type 2 diabetes. Here, we describe the *in vitro* characteristics and *in vivo* anti-diabetic efficacies of a novel GLP-1RA, termed SM102. The *in vitro* functions of SM102, including GLP-1R kinetic binding parameter, cAMP activation, endocytosis and recycling, were all evaluated using the INS-1 832/13 cells expressing human GLP-1R. Chronic efficacies study was performed to evaluate the effects of SM102 on the glycemic benefits, body weight loss and other diabetic complications in db/db mice. As a result, SM102 exhibited enhanced binding affinity and potency-driven bias in favor of cAMP over GLP-1R endocytosis and β-Arrestin 2 recruitment, as well as comparable insulin secretory response compared with Semaglutide. In addition, chronic treatment of SM102 led to more promising therapeutical effects on hyperglycemia, weight control and insulin resistance as well as dry eye syndrome (DES) than Semaglutide. Furthermore, SM102 could ameliorate diabetic DES via improving antioxidant properties, inflammatory factors and inhibiting MAPKs pathway in diabetic mice. In conclusion, SM102 is a G protein-biased agonist serving as a promising new GLP-1RA for treating diabetes and diabetic complications.

## Graphical abstract

**Figure:**
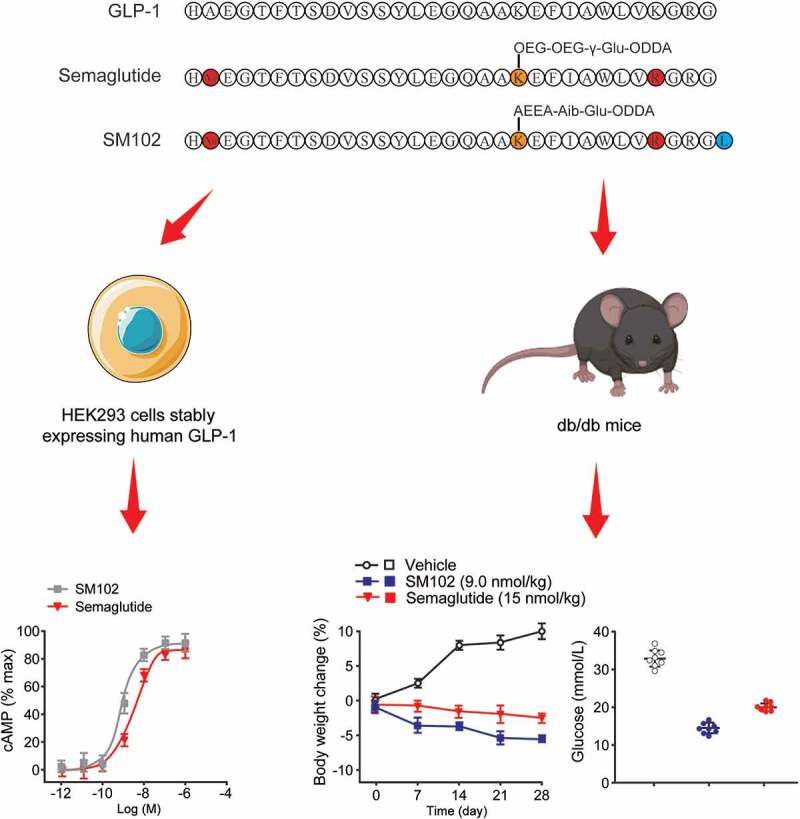

## 1. Introduction

Glucagon-like peptide-1 receptor agonists (GLP-1RAs) provide substantial reductions in HbA1c and significant body weight loss in patients with type 2 diabetes (T2D) and obesity [1,2]. GLP-1RAs are recommended as add-on therapy for patients not achieving target HbA1c on metformin, particularly in the context of cardiovascular disease or increased body mass index [3,4]. Six GLP-1RAs are FDA-approved for the treatment of patients with T2D, including short-acting, intermediate-acting, and long-acting therapies [5]. Circulatory half-life protraction of the latest generation of GLP-1RAs is achieved by fatty acid conjugation and resultant binding to albumin (Semaglutide), or by covalent linkage to Fc fragment of human IgG4 (dulaglutide), both of which increase effective molecular size sufficiently to virtually abolish clearance by renal filtration [6,7]. Glucose lowering by GLP-1RAs is due to a combination of effects, including the a direct action on pancreatic beta cell GLP-1Rs that potentiates glucose-stimulated insulin secretion, suppression of glucagon release, delay of gastric emptying, improvement on insulin sensitization due to weight loss, and neuronally mediated reductions in hepatic glucose output [8,9]. GLP-1RA treatment also exerts further beneficial metabolic effects such as improvements on lipid profiles and resolution of hepatic steatosis [10,11]. Cardiovascular events and all-cause mortality are also lowered in T2D [12]. Semaglutide is a GLP-1 analogue with 94% homology to human GLP-1. The long-term mechanism of Semaglutide is based on the modification of the structure to increase stabilization and avoid degradation by DPP-4 enzymes. Compared with the liraglutide of C16, the affinity of the growing carbon chain for albumin was enhanced by 5–6 times. It bound to albumin, increased the molecular weight of this product, avoided rapid renal clearance and prevented metabolic degradation, and prolonged the half-life in vivo. Lysine at position 34 was replaced with arginine to ensure stability of the C-side chain at position 26 with a half-life of up to 1 week. In clinical trials, Semaglutide significantly reduced HbAlc levels in T2D patients compared with sitagliptin, insulin glargine, and extended-release exenatide, respectively, demonstrating its significant hypoglycemic effect. In addition, it is able to reduce patient weight by reducing appetite and reducing food intake. Semaglutide also reduces the incidence of cardiovascular death, non-fatal myocardial infarction, or non-fatal stroke and becomes an antidiabetic drug with cardiovascular benefits.

As a Gαs-favoring G protein-coupled receptor (GPCR), many cellular actions of GLP-1RA activation are attributable to elevations in intracellular cyclic adenosine monophosphate (cAMP) [1,13]. However, recent studies have highlighted the complexities of GLP-1R signaling, including a possible role for Gαq, acting to mobilize intracellular Ca^2+^ as well as modulation of GLP-1R signal duration by β-arrestin-mediated desensitization and β-arrestin-independent endocytosis [14,15]. Ligand-specific preference for distinct GLP-1R-coupled cellular signaling and trafficking responses, or biased agonism, is a potential means to extend the duration of GLP-1RA action, typically through avoidance of receptor desensitization and downregulation [16,17].

In this study, we hypothesized that SM102, a novel long-acting, human GLP-1RA based on the sequence of native GLP-1 may trigger beneficial effects on diabetes and its complication in db/db mice. This agonist shares significant structural homology with the class-leading GLP-1RA Semaglutide (Ozempic^TM^) but with a distinct linker sequence between its polypeptide chain and albumin-binding acyl group, which could potentially affect its pharmacological properties. We evaluate the *in vitro* and *in vivo* potency of SM102 in comparison to the Semaglutide, a marketed GLP-1RA, using a variety of models, including cell lines, primary pancreatic islets and db/db mice, specifically aiming to identify evidence of biased agonism as a potential explanation for differences in therapeutic efficacy.

## 2. Materials and methods

### 2.1. Materials

SM102 was synthesized using solid-phase technology employing Fmoc amino acids and with suitable protections of other functional groups for coupling to grow the desired sequence [18]. Briefly, Rink amide resin (GyrosProtein Technologies Inc., Tucson USA.) was used as a solid support. Fmoc group removal was carried out through treatment with 20% 4-methylpiperidine in DMF (Aladdin, D112000). Subsequently, cleavage from the resin and deprotection of side chain functionalities were performed in one step using trifluoroacetic acid (TFA, Aladdin, T291771). A further Lys residue was acylated, with deprotection at appropriate stages and purification on preparative high performance liquid chromatography (HPLC) resulting in isolation of SM102.

### 2.2. Animals

Male C57BL6/J mice were purchased from Medical Experimental Center of Hebei University. Animals were housed in a pathogen-free facility with 12-hour light-dark cycle with free access to standard mouse chow diet and water in accordance with institutional guidelines for animal welfare at Medical Experimental Center of Hebei University. All *in vivo* animal experiments were approved by the Institutional Animal Ethics Committee of Hebei University with the approval code of SYXK002020-012.

### 2.3. Cell culture and functional assays

HEK293 cells stably expressing human GLP-1R (hGLP-1 R) (generated in-house using HEK293 cell line purchased from ATCC, Cat# CRL-1573) were maintained in Dulbecco’s Modified Eagle Medium (DMEM, Gibco, 11,054,020) with 10% fetal bovine serum (FBS, Gibco, 10,099,133 C), 1% penicillin/streptomycin (Gibco, 10,378,016) and 1 mg/mL G418 (Gibco, 10,131,035). Wild-type INS-1 832/3 cells, or INS-1 832/3 cells lacking endogenous GLP-1 R (generated in-house using INS-1 832/3 cell line purchased from NTCC, Cat# CVCL_ZL55) were maintained in RPMI-1640 (Gibco, 22,400,071) with 1 mM sodium pyruvate (Gibco, 11,360,070), 50 μM β-mercaptoethanol (Gibco, 21,985,023), 10% FBS, and 1% penicillin/streptomycin. PathHunter CHO-K1-βarr2-EAGLP1R cells (DiscoverX) were maintained in F12 medium (Gibco, 11,320,033) with 10% FBS, 1% penicillin/streptomycin, 1 mg/mL G418 and 250 μg/mL hygromycin (Gibco, 10,687,010).

HEK293 stably expressing hGLP-1 R cells were stimulated with agonist for 30 min at 37°C in serum-free medium (Gibco, 12,753,018). cAMP was determined at the end of the incubation period by homogenous time resolved fluorescence (HTRF; cAMP Dynamic 2 kit, Cisbio) using a Molecular Devices iD5 plate reader.

PathHunter CHO-K1-βarr2-EA-GLP-1 R cells were stimulated with agonist for 30 min at 37°C in Ham’s F12 medium (Gibco, 11,765,054) without FBS. At the end of the incubation period cells were lysed by addition of PathHunter detection reagents and luminescent signal was subsequently recorded from each well.

### 2.4. GLP-1 R recycling assay

INS-1 hGLP-1 R cells were seeded the day before the assay in 96-well imaging plates. Cells were washed twice with PBS, then stimulated with 100 nM of respective agonist in complete media, with an equal number of wells kept as vehicles. Following stimulation of the cells for 1 hour, they were washed twice with PBS and labeled with exendin-4-TMR13 for either 1 hour or 3 hours, washed again with PBS, and imaged via high content microscopy.

### 2.5. *Isolation and culture of mouse pancreatic islets and* in vitro *insulin secretion assays*

Mice were euthanized by cervical dislocation and pancreatic islets isolated by collagenase digestion as previously described and cultured in RPMI 1640 medium [19], 11 mM glucose, supplemented with 10% (v/v) FBS plus penicillin (100 units/mL), and streptomycin (0.1 mg/mL) at 37°C in an atmosphere of humidified air (95%) and CO_2_ (5%).

Mouse islets (10/well) were incubated in triplicate for each condition and treatment. Islets were preincubated for 1 hour in 3 mM glucose KRH buffer prior to secretion assay (30 min) in 3 mM or 11 mM glucose with vehicle or 100 nM SM102 or Semaglutide. The secretion medium was then collected to measure the insulin and proinsulin concentrations using an insulin HTRF kit (Cisbio Bioassays). INS-1 832/3 cells were treated with the indicated concentrations of SM102 or Semaglutide for 16 h in complete RPMI1640 medium at 11 mM glucose. A sample of supernatant was collected and analyzed for insulin content by HTRF.

### 2.6. Chronic administration study in db/db mice

The diabetic db/db mice (half male and half female, 8–12 weeks, body weight- 40-50 g) were brought from Cavens Company Ltd. (Changzhou) and housed in individual ventilated cages with free access to food and water and maintained on a 12-hour light/dark cycle. Animals were acclimatized for 3 days. On day 0, each animal was weighed using a digital weighing balance. Blood was collected by retro-orbital plexus puncture. Blood glucose level was measured using a hand-held blood glucose meter (Johnson & Johnson) and %HbA1c was measured using auto fluorescence immunoanalyzer.

Animals were divided into treatment groups (n = 8 per group; 4 male, 4 female) with matched baseline %HbA1c levels (mean 7.3%). Vehicle, SM102 (1, 3 and 9 nmol/kg), Semaglutide (15 nmol/kg) were subcutaneously injected into the neck region of the animals on every other day for 4 weeks.

Body weight and food intake were monitored serially. On day 29 of the study, 24 h following the last dose, blood was collected for measurement of %HbA1c, triglycerides, insulin, C-peptide and glucagon. The function of β-cell was assessed via calculating the HOMA-IR from blood concentrations of glucose and insulin.

### 2.7. Chronic administration study in diabetic DES mice

DES in db/db mice was induced by twice daily administration of 5 μL of 0.2% benzalkonium chloride (Sigma-Aldrich) for 7 days. All diabetic DES mice were divided into vehicle group, SM102 treatment groups (1, 3 and 9 nmol/kg), Semaglutide treatment group (15 nmol/kg), and dulaglutide (1.5 nmol/kg) (n = 8 per group; 4 male, 4 female). The drugs were injected subcutaneously into the neck region of the animals on every other day for 4 weeks.

The mouse’s lower eyelid was gently pulled downward, and a line with a 1 mm length of the phenol red cotton thread was placed on the upper part of the palpebral conjunctiva for 15 seconds near the inner part about one-third of the distance from the outer corner of the eyelid. The length of the colored part of the phenol red thread was recorded as the tear secretion. 1 μL of 0.1% liquid fluorescein sodium was instilled into the conjunctival sac followed by three blinks, and tear breakup time (BUT) was recorded in seconds.

At the end of treatment, animals were euthanized, and serum were isolated (12,000 rpm for 5 min at 4°C). The levels of interleukin-6 (IL-6), interleukin-1β (IL-1β), tumor necrosis factor-α (TNF-α), transforming growth factor β1 (TGF-β1) and malondialdehyde (MDA) as well as superoxide dismutase (SOD) activity in the supernatant were measured using appropriate ELISA kits brought from R&D company (USA). The expression levels of p38/p-p38, JNK/pJNK and ERK/p-ERK were analyzed by Western blot methods as previously reported [20].

Hematoxylin and eosin (H&E) staining were performed as described below: Corneas were fixed in 4% formalin. After dehydration, the specimens were embedded in paraffin and serial sections of 6 μm thickness were cut and stained with a hematoxylin-eosin reagent. Periodic Acid–Schiff (PAS) staining were performed as described below: Conjunctiva was fixed in 4% formalin. After dehydration, the specimens were embedded in paraffin and serial sections of 6 μm thickness were cut and stained with 0.5% PAS staining. The number of positively stained cup-shaped cells in the stained conjunctival sections was counted using image-Pro Plus6.0 (MediaCybernetics, USA).

### 2.8. Statistical analysis

Data are represented as mean ± standard error of the mean (SD), and the data processing and plotting was performed via the GraphPad Prism 8 software. Statistical comparisons were performed by Student’s t-test or one-way ANOVA as appropriate. Statistical significance was assigned where *p* < 0.05.

## 3. Results

In this study, we hypothesized that SM102, a novel long-acting, human GLP-1 RA based on the sequence of native GLP-1 may trigger beneficial effects on diabetes and its complication in db/db mice. This agonist shares significant structural homology with the class-leading GLP-1 RA Semaglutide (Ozempic^TM^) but with a distinct linker sequence between its polypeptide chain and albumin-binding acyl group, which could potentially affect its pharmacological properties. We evaluate the *in vitro* and *in vivo* potency of SM102 in comparison to the Semaglutide, a marketed GLP-1 RA, using a variety of models, including cell lines, primary pancreatic islets and db/db mice, specifically aiming to identify evidence of biased agonism as a potential explanation for differences in therapeutic efficacy.

### 3.1. SM102 exhibits increased G protein-biased agonism compared to Semaglutide

**Figure 1.**
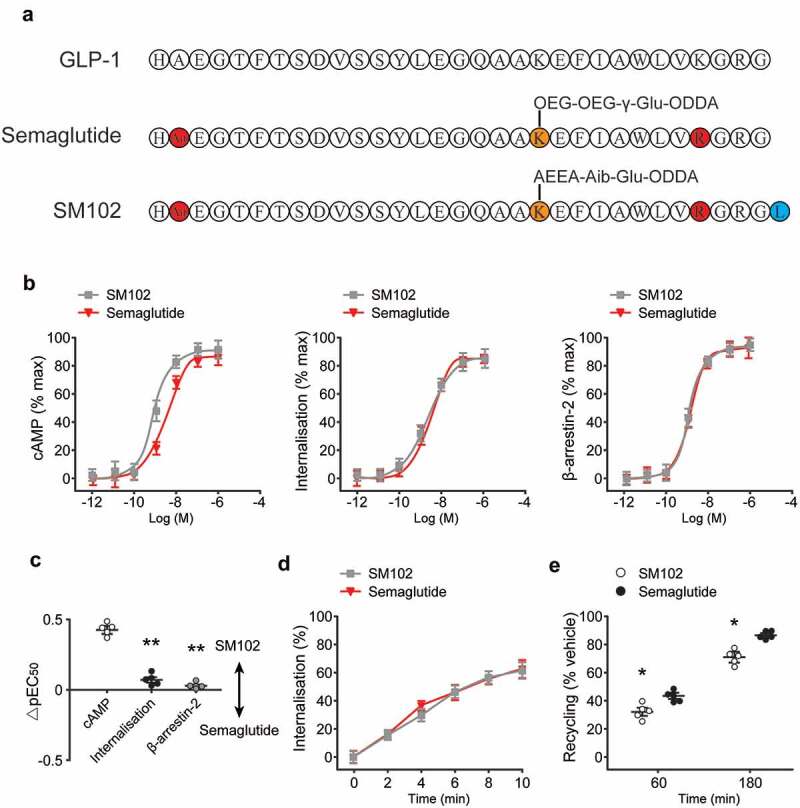
*In vitro* pharmacological characterization of SM102 in comparison to Semaglutide. (a) Amino acid sequences of GLP-1(7–37), Semaglutide and SM102. (b) cAMP production, GLP-1 R internalization and β-arrestin-2 recruitment. (c) Subtracted pEC50 estimates from (B), compared by one-way ANOVA with Tukey’s test. (d) Quantification of internalized GLP-1 R. (e) Quantification of exendin-4-TMR uptake after prior treatment with 100 nM Semaglutide or SM102, indicating reemergence of internalized GLP-1 R over time. Data represented as mean ± SD (n = 5), *^,^ **p < 0.05, 0.01.

The structure of SM102 is shown in Figure 1(a) alongside that of native GLP-1(7–37) and the current class-leading GLP-1 RA, Semaglutide. Similar to Semaglutide, SM102 differs from GLP-1 by the substitution of the native second position amino acid alanine with Aib, confering resistance to cleavage by DPP-4. Both SM102 and Semaglutide also feature a substitution of Lysine→Arginine at position 28, allowing the site-specific acylation at the remaining lysine at position 20. SM102 also has an additional C-terminal leucine, not found in Semaglutide, and a distinct linker sequence that couples the peptide to an octadecanedioic acid albumin-binding moiety. Specifically, the linker fragment includes Aib to provide stability toward peptidases and to balance hydrogen bonding donor and acceptor properties in the whole side chain with acyl group.

To determine how the above structural differences between SM102 and Semaglutide might affect ligand pharmacology, the cAMP accumulation and GLP-1 R endocytosis were first assessed, the latter using a high content imaging-based assay, which further confirmed that signaling potency was increased without a corresponding increase in internalization (Figure 1(b)). The β-arrestin-2 recruitment responses with both ligands were also measured, which showed no differences in response potency or E_max_. Comparison of potency differences within each assay confirmed significant signaling selectivity in favor of cAMP for SM102 compared to endocytosis and β-arrestin-2 recruitment (Figure 1(c)).

Both ligands were able to induce rapid GLP-1 R endocytosis (Figure 1(d)), with no clear difference in kinetics observed between the two. GLP-1 R recycling was also determined by incubating cells with fluorescent exendin-4-TMR after an initial internalization step, with measured fluorescence uptake indicative of replenishment of plasma membrane GLP-1 R after Semaglutide or SM102 pre-treatment and washout. This approach revealed modestly reduced recycling with SM102, which is compatible with previous evidence showing that lower GLP-1 RA dissociation rates are associated with slower GLP-1 R recycling (Figure 1(e)). Overall, these data reveal moderate increases in GLP-1 R binding affinity and cAMP signaling for SM102 compared to Semaglutide, without a corresponding increase in receptor endocytosis. Therefore, SM102 shows G protein-biased agonism relative to Semaglutide.

### 3.2. Insulin secretion test in mouse islets

**Figure 2.**
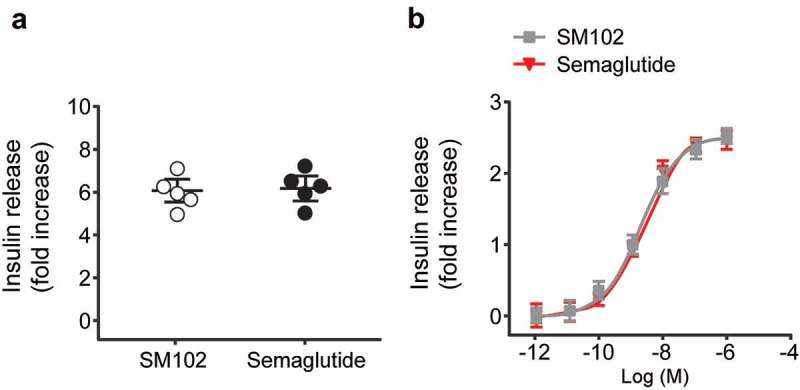
Comparable *in vitro* insulin secretory effects of SM102 and Semaglutide. Insulin secretion from (a) mouse islets and (b) INS-1 832/3 cells. Data represented as mean ± SD (n = 5), *^,^ **p < 0.05, 0.01.

Static acute insulin secretion experiments was performed using the islets from C57BL/6 mice at 11 mM glucose, and the results demonstrated that the potentiation of GSIS by both Semaglutide and SM102 at 100 nM concentration, with no difference between the effect of either agonist (Figure 2(a)). In case SM102 and Semaglutide differed in their ability to desensitize the GLP-1 R, the insulin release after overnight incubation of INS-1 832/3 cells with a range of concentrations of each agonist were also measured, and results showed that the responses were virtually indistinguishable between the two compounds (Figure 2(b)). These data demonstrate that, as expected, SM102 is capable of potentiating insulin release at elevated glucose concentrations, but do not provide evidence of an increased insulinotropic effect of SM102 compared to Semaglutide.

### 3.3. Long lasting and potent anti-diabetic effects of SM102 in db/db mice

**Figure 3.**
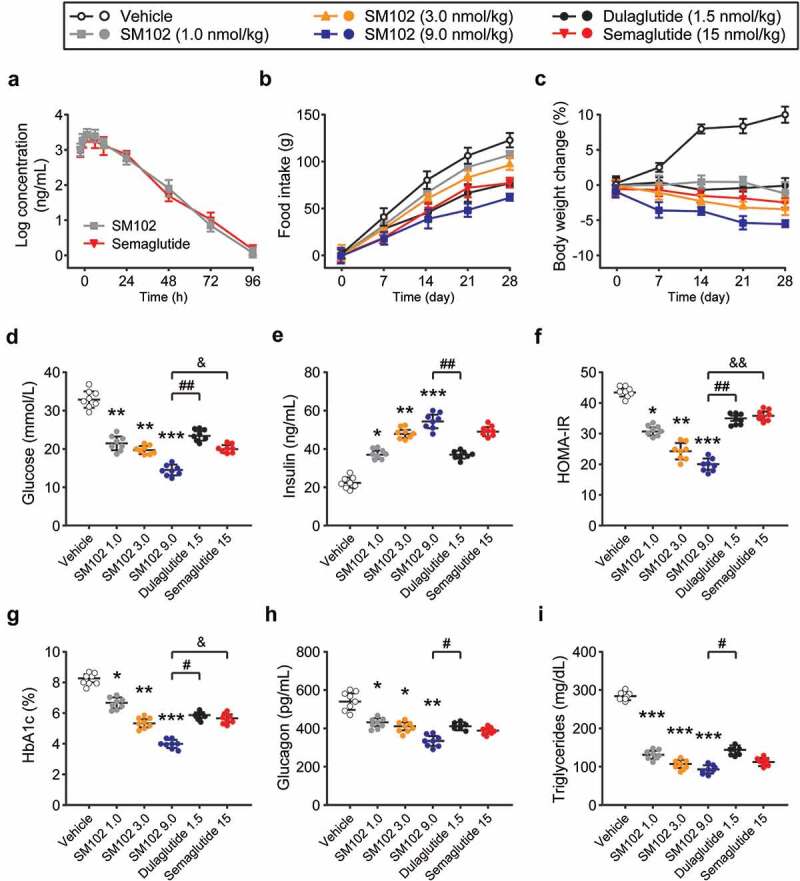
SM102 has powerful anti-diabetic effects *in vivo*. (a) Plasma concentration of SM102 and Semaglutide following single subcutaneous injection (1 mg/kg) in male CD-1 mice. (b) Cumulative food intake and (c) body weight change. (d) Plasma glucose concentration, (e) insulin concentration, HOMA-IR level, HbA1c%, plasma glucagon concentration and plasma triglyceride concentration at the end of the study. *^,^ **^,^ ***p < 0.05, 0.01, 0.001 versus vehicle, ^#, ##^ *p* < 0.05, 0.01 versus dulaglutide, ^&^ *p* < 0.05 versus Semaglutide. Data represented as mean ± SD (n = 8).

The pharmacokinetics of SM102 were evaluated in CD-1 mice (Figure 3(a)). Plasma half-life of SM-102 after subcutaneous injection was ~47.65 h, which is comparable to Semaglutide at same dose. Chronic administration study in db/db mice with three separate dosing regimens of SM102, as well as Semaglutide and dulaglutide, were furtherly performed. These mice were injected s.c. every other day for 28 days with 1, 3, or 9 nmol/kg SM102. Semaglutide was dosed at 15 nmol/kg every other day, providing dose equivalence to a maximally effective dose in mice. Dulaglutide, another commonly used one weekly injectable GLP-1 RA, was dosed every other day at 1.5 nmol/kg as an additional comparator. All ligand doses tested led to marked improvements in important metabolic readouts compared to vehicle control, including the reduced food intake, body weight, glucose, HbA1c, plasma glucagon and triglyceride levels, increased insulin levels, and enhanced beta cell function as measured by HOMA-IR (Figure 3(b-i)). It is worth noting that insulin release in cells treated with SM102 shows a similar profile compared to Semaglutide, which suggested the significant decreased insulin clearance in db/db mice after SM102 treatment. Notably, the therapeutic effect of 3 nmol/kg of SM102 was comparable to that of 4 times higher doses of somalutin, and the therapeutic effect of 9 nmol/kg of SM102 was superior, including weight loss (Figure 3(c)), glucose (Figure 3(d)) and HOMA-IR (Figure 3(g)). SM102 also outperformed dulaglutide. Therefore, the above results suggest that SM102 holds desirable antidiabetic properties.

### 3.4. Chronic treatment of SM102 improves the damage of cornea and conjunctiva in diabetic DES mice

**Figure 4.**
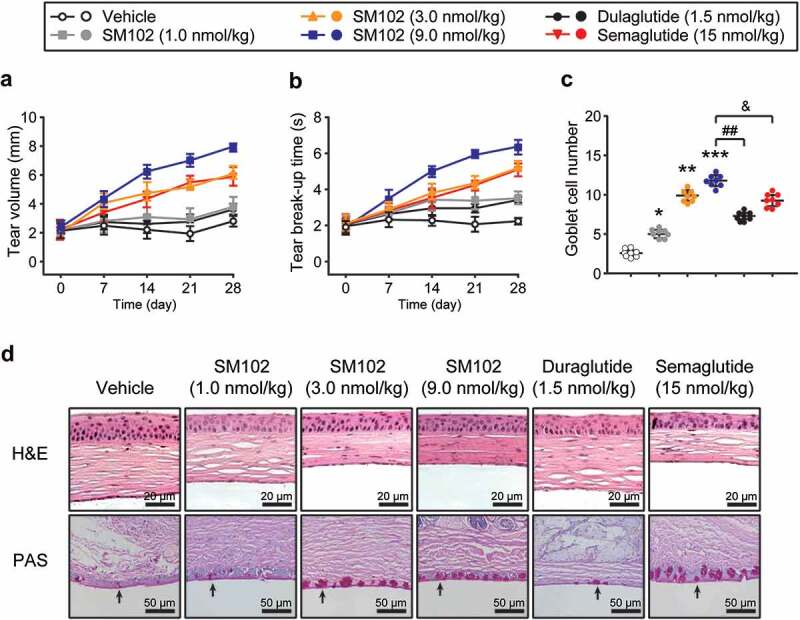
Protective effects of SM102 on diabetic DES after 4-week treatment. (a) Tear volume and (b) breakup time of tear film in diabetic DES mice during 4-week SM102 treatment. (c) The number of conjunctival goblet cells in diabetic DES mice after 4-week SM102 treatment. (d) Pathological morphology of cornea or conjunctiva in diabetic DES mice presenting by H&E staining (top row) or PAS staining (bottom row). *^,^ **^,^ ***p < 0.05, 0.01, 0.001 versus vehicle, ^##^ p < 0.01 versus dulaglutide, ^&^ *p* < 0.05 versus Semaglutide by indicated statistical test. Data represented as mean ± SD (n = 8).

Tear secretion and breakup time of tear film are two key indicators for evaluating dry eye symptoms [21]. First, the volume of tear secretion by measuring the moist length of phenol red cotton lines were determined. As shown in Figure 4(a), diabetic DES mice in control group had a low basal tear secretion throughout the treatment. After 4 weeks of continuous treatment with SM102, the tear secretion level was significantly enhanced compared to control group. Interestingly, mice treated with 9 nmol/kg SM102 had significantly higher tear secretion levels than those treated with Semaglutide or dulaglutide. In addition, SM102 treatment significantly prolonged breakup time of tear film in mice, illustrating a significant enhancement in tear film stability (Figure 4(b)). The structural changes of corneal tissue were observed by H&E staining and the results in Figure 4(d) shows that in the control group, the cornea was thickened, and then the thickness of the epithelial cell layer was increased with the irregular arrangement of epithelial cells and stromal layer fibrocytes. In contrast, corneal epithelial cells were closely packed and well-stratified in the medium and high dose SM102 treatment groups, indicating that SM102 significantly reversed corneal tissue damage. In addition, the results of PAS staining of conjunctival tissue showed that the number of conjunctival goblet cells was significantly reduced in diabetic DES mice, and the cell morphology exhibited a small and atrophic morphology. Compared with control mice, the number of conjunctival goblet cells in the 1, 3 and 9 nmol/kg SM102-treated groups increased approximately 1-, 4-, and 5-fold, respectively (Figure 4(c)). In addition, the SM102-treated group showed a statistically significant difference compared with the Semaglutide-treated or dulaglutide-treated group.

### 3.5. SM102 ameliorates diabetic DES via improving antioxidant properties, inflammatory factors and inhibiting MAPKs pathways

Development of diabetic DES is associated with oxidative stress and inflammation. The intrinsic antioxidant activity as well as inflammation-related factors in the corneas of control and SM102-treated mice were further compared. The concentration of MDA in corneal homogenate was significantly decreased in SM102 group compared with that of vehicle group. Meanwhile, the SOD activity in SM102-treated group presented a dose-dependent significant increase within the dose range of 1 ~ 9 nmol/kg, indicating that 4 weeks of SM102 treatment significantly improved the antioxidant protective effect of the cornea in diabetic DES mice. In addition, SM102 treatment significantly reduced the levels of pro-inflammatory factors, including IL-6, IL-1β, and TNF-α, and increased the levels of TGF-β1 in corneal homogenates compared with those of the control mice. Overall, the protective effect of SM102 on diabetic DES may involve inhibiting the inflammatory response of the ocular surface by inhibiting previous oxidative stress and/or by specifically hyper-stimulating the secretion of the cytokine TGF-β1.

**Figure 5.**
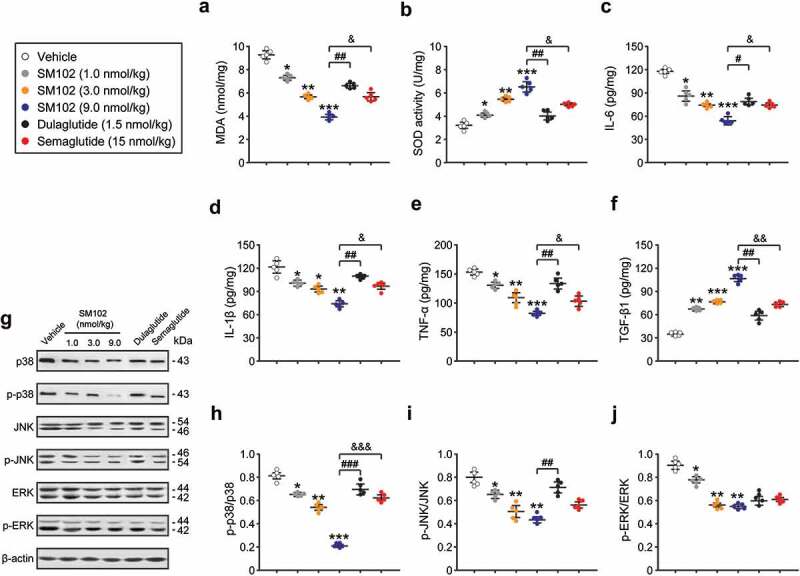
SM102 improve oxidative stress and inflammation responses via inhibiting MAPKs pathway. (a-b) The MDA level and SOD activity, and (c-f) the levels of IL-6, IL-1β, TNF-α and TGF-β1 in serum of diabetic mice after 4-week SM102 treatment. (g) Western blot image and (h-j) quantitative analysis of p-p38/p38, p-JNK/JNK and p-ERK/ERK in diabetic mice after 4-week SM102 treatment. *^,^ **^,^ ***p < 0.05, 0.01, 0.001 versus vehicle, ^#, ##, ###^ *p* < 0.05, 0.01, 0.001 versus dulaglutide, ^&, &&, &&&^ *p* < 0.05, 0.01, 0.001 versus Semaglutide. Data represented as mean ± SD (n = 8).

The activation of mitogen-activated protein kinases (MAPKs) signaling pathway has a close link with the development of inflammation. Oxidative stress can activate the MAPKs signaling pathway to induce the production of inflammatory cytokines [22]. To validate the involvement of MAPKs signaling pathways in the development of inflammation, we first examined the phosphorylation levels of MAPKs by Western blotting mehtods. As shown in Figure 5(g-j), treatment with all 3 doses of SM102 resulted in significantly lower phosphorylation levels of p38, JNK and ERK compared with the model group (all *p* < 0.05). Overall, the MAPKs pathway is involved in the development of corneal oxidative stress and inflammation. Moreover, long-term treatment with SM102 prevented the excessive phosphorylation of p38, JNK and ERK MAPK, thereby inhibiting the development of inflammation and oxidative stress in diabetic DES mice.

## 4. Discussion

In current study, we described the *in vitro* and *in vivo* evaluation of SM102, a novel long-lasting GLP-1 RA with partial sequence homology to Semaglutide. Although SM102 was administered at a relatively lower dose, it was more effective than semaglutide in lowering blood glucose in db/db mice and exhibited a range of other beneficial metabolic effects. These findings thus highlight the promise of this novel agent for the treatment of T2D. Notably, our findings seem likely to be related to the higher binding affinity of SM102 and biased agonism of G proteins.

SM102 shares substantial structural similarities with Semaglutide, including a DPP-4 resistance-conferring alanine → Aib substitution at position 2, as well as an octadecanedioic acid group to promote reversible albumin binding. These similarities likely account for the very similar pharmacokinetic properties we observed with SM102 compared to publicly available data for Semaglutide. However, the linker strategy used to couple acyl moiety to peptide differs between SM102 and Semaglutide, and this may account for the *in vitro* pharmacological differences between the two agents, including increased GLP-1 R binding affinity with SM102. During the course of the development of Semaglutide, a number of data demonstrated that different side chain linkers may have major effects on GLP-1 R binding, with a greater than 1000-fold reduction in cAMP signaling potency in some cases [23]. In conclusion, the novel linker sequence of SM102 facilitates improvements in GLP-1 R binding without leading to an unacceptable reduction in pharmacokinetics.

Previous reports demonstrated how G protein-selective biased agonism is associated with profound alterations to GLP-1 R trafficking, resulting in improved insulinotropic and anti-diabetic effects through avoidance of GLP-1 R desensitization and downregulation [24,25]. The majority of these examples showed ‘efficacy-dominant’ G protein bias, in which reductions in maximum obtainable GLP-1 R endocytosis or β-arrestin recruitment appeared necessary to drive the improvements in sustained duration of action [26,27].

Interestingly, most efficacy-driven G protein-biased agonists showed moderately reduced efficacy for coupling to G protein signal, but signal amplification within the Gαs/cAMP/PKA pathway allowed for full downstream responses, ultimately leading to paradoxically increased efficacy due to reduced desensitization [28]. In contrast, another G protein-biased GLP-1 R (‘P5’) shows more modest reductions transducer coupling efficacy, smaller changes in GLP-1 R desensitization and downregulation, and does not show enhanced insulinotropic efficacy *in vitro* or *in vivo*. The cAMP-favoring bias observed with SM102 clearly resulted from increased cAMP potency compared to Semaglutide, rather than via reduced efficacy for endocytosis or β-arrestin-2 recruitment. In keeping with the paradigm of efficacy-driven bias being required for augmentation of insulin secretion, SM102 showed no enhancement in sustained insulin secretion *in vitro*. As a further consideration, it is notable that GLP-1 R recycling with SM102 was subtly delayed compared to with Semaglutide treatment, which would be expected to mitigate against any hypothetical increases in sustained insulin secretion by restricting the availability of surface GLP-1 R during continuous exposure.

SM102 showed potent antidiabetic effect in db/db mice. Even at an almost 10-fold lower dose (1 nmol/kg vs 15 nmol/kg), improvement in blood glucose and insulin secretion after 4 weeks of treatment with SM102 was comparable to Semaglutide. At the highest SM102 dose tested (9 nmol/kg), which is still under half that of Semaglutide, blood glucose-lowering and HOMA-estimated beta cell function were significantly better, with trend toward greater effects on HbA1c and suppression of plasma glucagon. It is important to highlight that 9 nmol/kg SM102 also led to greater body weight loss than Semaglutide. Additional weight loss is a likely explanation for the enhanced effects of SM102 that does not require agonist-specific differences in direct action on the islet, particularly in the absence of any measured differences in acute or sustained insulin secretion between agonists in our *in vitro* studies. Notably, the middle dose of SM102 tested (3 nmol/kg) showed very similar effects on body weight to Semaglutide, and almost identical effects on other metabolic parameters.

Our study has several limitations. We performed most of our pharmacological evaluations using human GLP-1 R-expressing systems, whereas it could be argued that a link to *in vivo* effects would be better represented by mouse GLP-1 R studies. On the other hand, orthosteric GLP-1 R biased agonist effects tend to be similar at rodent and human GLP-1 R, and establishing the pharmacology in a human system is more translationally relevant [16]. We focused in the study on Gαs-dependent cAMP signaling on the basis that this is the primary Gα subtype coupled to GLP-1 R activation; however, a recent study has elevated the possibility of a role for Gαq-dependent signaling, which is a subject for future investigation [28]. We did not directly compare the albumin binding properties of SM102 and Semaglutide, which could differ due to the different linker sequence, despite the same C18 acyl diacid in both molecules. On the other hand, the seemingly identical kinetic properties of SM102 and Semaglutide argue for a major difference in albumin binding. We also note that while we evaluated insulin secretion over a wide range of agonist concentrations in clonal INS-1 832/13 β-cells, in primary mouse and human islets, our data are limited to the effect of high doses of the drug, which may exceed that observed after *in vivo* injection.

However, in a more limited number of experiments using lower doses (1 and 10 nM) differences were not apparent between the actions on glucose-stimulated insulin secretion of SM102 and Semaglutide (data not shown). Finally, our main *in vivo* study was primarily aimed at establishing the therapeutic effects of repeated SM102 administration, meaning we were unable to assess effects in the absence of weight loss. However, by using different SM102 dosing regimens, we could compare SM102 against comparator GLP-1 RAs at equivalent weight loss. Furthermore, this novel drug SM102 might perform relative to others that were not tested here, such as Liraglutide. Liraglutide is FDA approved antidiabetic agent that requires once-daily injection. Interestingly, administration of SM102 every other day in db/db mice resulted in a desirable antidiabetic effect. Therefore, we boldly guess that SM102 is more promising than Liraglutide in treating diabetes.

Diabetes mellitus is one of the risk factors for the onset of eye diseases. Persistent hyperglycemia causes abnormal glucose metabolism and accumulation of advanced glycation end products in ocular surface cells, which causes ocular surface inflammation and damage. Expression of SIRTI and development of oxidative stress may also induce the diabetic dry eye (DES). Interestingly, a very good therapeutic effect by applying SM-102 to the existing dry eye model of our group were found. As the results exhibited in present study, the therapeutic effects of SM102 on diabetic dry eye was demonstrated *in vivo* in an animal model with diabetic DES. Treatment of SM102 significantly increased tear production and breakup time of tear film in diabetic DES mice, which could be the basis of SM102 treatment on diabetic DES. Furthermore, chronic treatment with SM102 significantly improved the morphological characteristics of corneal and conjunctival tissues and reversed the damage of conjunctival goblet cells in diabetic DES mice. Not only that, SM102 treatment significantly reduced oxidative stress and inflammation development in the ocular surface of diabetic DES mice by inhibiting the phosphorylation of MAPKs signaling pathway. The above results indicate that SM102 is expected to be a therapeutic candidate for diabetic dry eye.

## 5. Conclusion

In conclusion, SM102 is a G protein-biased agonist that shows powerful anti-diabetic effects in db/db mice, and may serve as a promising new GLP-1 RA for with T2D patients with complications.

## Highlights

- SM102 exhibits increased G protein-biased agonism compared to Semaglutide
- SM102 is capable of potentiating insulin release at elevated glucose concentrations
- SM102 inhibited inflammation and oxidative stress via MAPKs pathways
- SM102 holds desirable properties for diabetes and its complications
